# Quantitative Analyses Help in Choosing Between Simultaneous vs. Separate EEG and fMRI

**DOI:** 10.3389/fnins.2018.01009

**Published:** 2019-01-10

**Authors:** Maarten Schrooten, Rik Vandenberghe, Ronald Peeters, Patrick Dupont

**Affiliations:** ^1^Laboratory for Cognitive Neurology, KU Leuven, Leuven, Belgium; ^2^Department of Neurology, University Hospitals Leuven, Leuven, Belgium; ^3^Department of Radiology, University Hospitals Leuven, Leuven, Belgium

**Keywords:** simultaneous, EEG-fMRI, quality, artifacts, TSNR, FFT, spectrogram

## Abstract

Simultaneous registration of scalp electroencephalography (EEG) and functional magnetic resonance imaging (fMRI) is considered an attractive approach for studying brain function non-invasively. It combines the better spatial resolution of fMRI with the better temporal resolution of EEG, but comes at the cost of increased measurement artifact and the accompanying artifact preprocessing. This paper presents a study of the residual signal quality based on temporal signal to noise ratio (TSNR) for fMRI and fast Fourier transform (FFT) for EEG, after optimized conventional signal preprocessing. Measurements outside the magnetic resonance imaging scanner and inside the scanner prior to and during image acquisition were compared. For EEG, frequency and region dependent significant effects on FFT squared amplitudes were observed between separately vs. simultaneously recorded EEG and fMRI, with larger effects during image acquisition than without image acquisition inside the scanner bore. A graphical user interface was developed to aid in quality checking these measurements. For fMRI, separately recorded EEG-fMRI revealed relatively large areas with a significantly higher TSNR in right occipital and parietal regions and in the cingulum, compared to separately recorded EEG-fMRI. Simultaneously recorded EEG-fMRI showed significantly higher TSNR in inferior occipital cortex, diencephalon and brainstem, compared to separately recorded EEG-fMRI. Quantification of EEG and fMRI signals showed significant, but sometimes subtle, changes between separate compared to simultaneous EEG-fMRI measurements. To avoid interference with the experiment of interest in a simultaneous EEG-fMRI measurement, it seems warranted to perform a quantitative evaluation to ensure that there are no such uncorrectable effects present in regions or frequencies that are of special interest to the research question at hand.

## 1. Introduction

Simultaneous registration of scalp EEG and fMRI is considered an attractive approach for studying brain function non-invasively, because it combines the better spatial resolution of fMRI with the better temporal resolution of EEG (Laufs, 2012; Jorge et al., 2014; Murta et al., 2015; Abreu et al., 2018).

The EEG and fMRI signal sources are different. The most used fMRI measure, is the change in the blood oxygen level-dependent (BOLD) contrast, reflecting physiological changes of cortical and subcortical neuronal activity (Ogawa et al., 2000). EEG, however, mainly measures the summed post-synaptic, radially oriented, cortical electrical neuronal activity and is thus a more direct measurement of neural activity (Mulert and Lemieux, 2010). Tangential and (deeper) subcortical sources typically do not contribute to EEG to a substantial extent.

Simultaneous registration is especially useful for the analysis of single trial correlations between the fMRI and EEG signal (Jorge et al., 2015). The validity of this combined approach relies on the tight coupling of the hemodynamic and electrophysiological responses in selected conditions (Huster et al., 2012). It has been successfully used in several fields of fundamental research (e.g., Mijović et al., 2012) as well as in clinical practice (e.g., Tousseyn et al., 2014). However, simultaneous registration does come at the cost of increased measurement artifact and the accompanying artifact preprocessing, especially for EEG (Huster et al., 2012).

We acquired two datasets studying the same paradigm: one where the EEG and fMRI data were acquired separately and one where the EEG and fMRI data were acquired simultaneously. While not planned as an a priori head to head comparison, five subjects participated in both experiments. Even though the sample size is limited, we report the results of analyses of the residual signal quality after optimized conventional signal preprocessing for task-related fMRI and EEG during an attention task measured separately and simultaneously in the same subjects. Since we always perform a short EEG measurement at rest outside the scanner and inside the scanner before image acquisition, additional EEG signal quality could be investigated to interpret some of the results. The strength of our dataset compared to previous studies, is that it consists of the same subjects in both conditions, that data inside and outside the scanner was recorded and that we present a quantitative rather than a qualitative study method.

## 2. Materials and Methods

### 2.1. Subjects

Five healthy subjects participated twice in the same covert attention task: once with EEG and fMRI data acquired in separate sessions ([EEG][fMRI]) and once with EEG and fMRI data acquired in a simultaneous session ([EEG fMRI]). All five subjects were European Caucasian and between the ages of 23 and 25 years. Four were female. Participants did not have a personal or family history of a neurological, psychiatric or ophthalmological disorder, nor were taking any neurotropic drugs. All subjects performed the experiment in the [EEG][fMRI] recording condition first (with an average interval between EEG measurements of the separate and simultaneous recordings of 380 days; range 239–560 days). In the [EEG][fMRI] experiment, two subjects performed the EEG part first (with an average interval between EEG and fMRI measurements of 153 days; range 151–155 days) and three subjects performed the fMRI part first (with an average interval between both measurements of 27 days; range 8–39 days). In the MRI scanner, functional scans were always acquired before the structural scan.

Task datasets contain epochs with a 200 ms symbolic cue, a 280 ms or 380 ms delay, a 200 ms stimulus consisting of a fixation cross and a unilateral or bilateral greyscale sinusoid gratings and a 1,650 ms response window (Schrooten et al., 2017). Subjects had to judge whether the greyscale grating, indicated by the symbolic cue, was slightly rotated clockwise or anticlockwise relative to a 45° clockwise-rotated reference grating. The degree of difficulty was tailored to the subject to ensure a high attentional load.

Subjects were recruited through advertisements among university students from the Biomedical Sciences group of the University of Leuven and hospital personnel of the University Hospitals Leuven. Experiments were approved by the Ethics Committee of the University Hospitals Leuven (S51126 & S56814) and were performed according to the World Medical Association Declaration of Helsinki (Association, 2013). All subjects provided written informed consent.

### 2.2. EEG

#### 2.2.1. EEG Recording

In the [EEG][fMRI]^1^ recording condition, EEG acquisition was performed in an unshielded room with a WaveGuard 64-channel EEG cap, following the 10–5 system (Oostenveld and Praamstra, 2001) of electrode placement, a 64-channel asalab amplifier at a sampling frequency of 1,024 Hz and a resolution of 22 bit, with active shielded micro coax EEG channels and the Advanced Source Analysis (ASA) software. During all measurements, impedances for all electrodes were kept below 10 kΩ and in the same range. Data were acquired with AFz as common hardware reference and were stored with the linked mastoid electrodes as reference. No hardware bandpass filtering was applied. Data was imported into EEGLAB with the ANTeepimport plugin. For one subject two electrodes (AF7, FT7) were clearly of too low quality by visual inspection (drift, high frequency noise). These were spherically interpolated in EEGLAB. Stimuli were presented on a Dell 1707FPt display (Dell Technologies, Round Rock, TX, United States) at a refresh rate of 60 Hz. The recording room ventilation system was not interrupted during acquisition.

In the [EEG fMRI] recording condition EEG acquisition was performed with a BrainCap MR 64-channel EEG cap, following the 10–5 system of electrode placement, a BrainAmp MR amplifier at a sampling frequency of 5,000 Hz, with a resolution of 16 bit, 0.016–250 Hz hardware bandpass filtering and BrainVision Recorder software, largely adhering to the advices formulated in Mullinger et al. (2013). Synchronization between the magnetic resonance imaging (MRI) and the EEG clock was in place. During all measurements, impedances for all electrodes were kept below 10 kΩ. Data were acquired and stored with FCz as common hardware reference. Data was imported into EEGLAB with the bvaio plugin. Recordings were made during the experimental task ([EEG fMRI]), inside the scanner during rest before the task and before image acquisition ([EEG fMRI]_pre−acquisition_) and during rest before entering the scanner room ([EEG fMRI]_outside_). Our recording system has two amplifiers (32 channels each; channels for amplifier 1: Fp1/2, F3/4, C3/4, P3/4, O1/O2, F7/8, T7/8, P7/8, Fz, Cz, Pz, Oz, FC1/2, CP1/2, FC5/6, CP5/6, CP9/10, EOG, ECG; channels for amplifier 2: F1/2, C1/2, P1/2, AF3/4, FC3/4, CP3/4, PO3/4, F5/6, C5/6, P5/6, AF7/8, FT7/8, TP7/8, PO7/8, Fpz, AFz, CPz, POz). The amplifiers were placed on top of each other inside the magnet every time in the same order. The helium pump was switched off during fMRI acquisition (a default setting of the used fMRI recording equipment), but not before. Lighting was switched off and stimuli were presented via a coil mirror on an Invivo Esys display (Inivo Corporation, Gainesville, FL, United States) at a refresh rate of 60 Hz. Ventilation was not interrupted. Wires were immobilized with sandbags.

#### 2.2.2. EEG Preprocessing

For all EEG measurements during fMRI acquisition, gradient artifact was removed before any other processing, using the weighted moving averages of artifact epochs technique (Allen et al., 2000) as implemented in the Bergen fMRI toolbox. Twenty-five artifact epochs per average were used. Since subjects essentially did not move throughout the experiment, realignment parameter-informed artifact correction (Moosmann et al., 2009) provided no benefit. For all other EEG measurements this preprocessing step didn't apply.

Data were resampled to 500 Hz and bandpass filtered with a 2nd order infinite impulse response Butterworth filter between 0.5 and 30 Hz (Miller et al., 2015) as implemented in ERPLAB.

For all EEG measurements inside the MRI scanner, QRS complexes were marked using an in-house developed, semi-automatic, supervised method after the removal of the gradient artifact and bandpass filtering. The EEG signal was decomposed into independent components using fast independent component analysis. For every component a time-amplitude signal was reconstructed, and independent components best capturing the QRS complex were manually selected. A reconstructed EEG signal based on these manually selected independent components was used solely to determine QRS peak latencies. Peaks were searched with the MATLAB's findpeaks function using a minimal peak distance based on a presumed heart rate below 110 beats per minute. Peak search was further refined with a manually defined minimal peak prominence based on a histogram of the peak prominences from the previous step. The semi-automatically found QRS peaks were all visually inspected and marks were manually corrected via a graphical user interface where needed. The found peak latencies were back ported to the bandpass filtered preprocessed original EEG and subsequently, pulse artifact subtraction was done with the FMRIB toolbox using a Gaussian-weighted mean template (Niazy et al., 2005). For EEG measurements outside the MRI scanner, this preprocessing step didn't apply.

Data were re-referenced to the average reference using EEGLAB. Channels not shared between the datasets were removed (PO5, PO6, AFz, non-EEG-channels), resulting in 60 (alphabetically sorted) remaining electrodes (AF3/4, AF7/8, C1/2, C3/4, C5/6, CP1/2, CP3/4, CP5/6, CPz, Cz, F1/2, F3/4, F5/6, F7/8, FC1/2, FC3/4, FC5/6, FCz, FT7/8, Fp1/2, Fpz, Fz, O1/2, Oz, P1/2, P3/4, P5/6, P7/8, PO3/4, PO7/8, POz, Pz, T7/8, TP7/8) common to all recording conditions.

All EEG data recorded during the covert attention task was epoched relative to stimulus onset (–0.8 to 1.8 s) and baseline corrected using the pre-cue interval (–0.8 to –0.6 s). We chose the pre-cue interval as a baseline, since the cue–stimulus interval was not strictly identical for all epochs. This choice better reflects a true baseline.

Uncontrolled variability is present in the main EEG analysis (the complete EEG acquisition system, including amplifier and cap hardware are different for the [EEG][fMRI] and [EEG fMRI] conditions). To further investigate whether the effects of the main analysis are purely attributable to these differences, EEG fragments recorded before the task were additionally analyzed (in the [EEG fMRI]_outside_ and [EEG fMRI]_pre−acquisition_ recording condition). These fragments were intentionally recorded for live visual inspection of recording quality. Subjects were instructed to relax, have their eyes open, and to restrict eye movements. The recording length was not standardized. Continuous EEG segments free of artifacts (such as movement artifact, muscle artifact and excessive blinking) were used (median length 46 s, range 13–144 s).

Per EEG channel, squared amplitudes of FFT output for the frequency (f) range 0–65 Hz were calculated with ERPLAB's fourieeg-function. This will be denoted by *A*^2^(*f*). We chose to analyze up to 65 Hz as the bandpass gain curve somewhat flattens after this frequency. Electrical interference is expected in this higher frequency range, possibly allowing us to identify artifacts in lower frequency ranges as subharmonics of higher frequencies. Mean squared amplitudes of FFT were calculated around every integer (center) frequency between 1 and 64 Hz using a frequency span of 2 Hz. We chose to use FFT to assess data quality, since it is free of a priori assumptions about the data and it is a commonly used measure in EEG analyses.

Paired *t*-tests of the index of dispersion for the aforementioned mean squared amplitudes per frequency were performed to compare the [EEG][fMRI] and the [EEG fMRI] recording conditions, in search of frequency dependent differences. Index of dispersion was defined as the variance over all electrodes divided by the mean of all electrodes. The statistical threshold was set at a false discovery rate (FDR) corrected *p* < 0.05.

**Figure 1:**
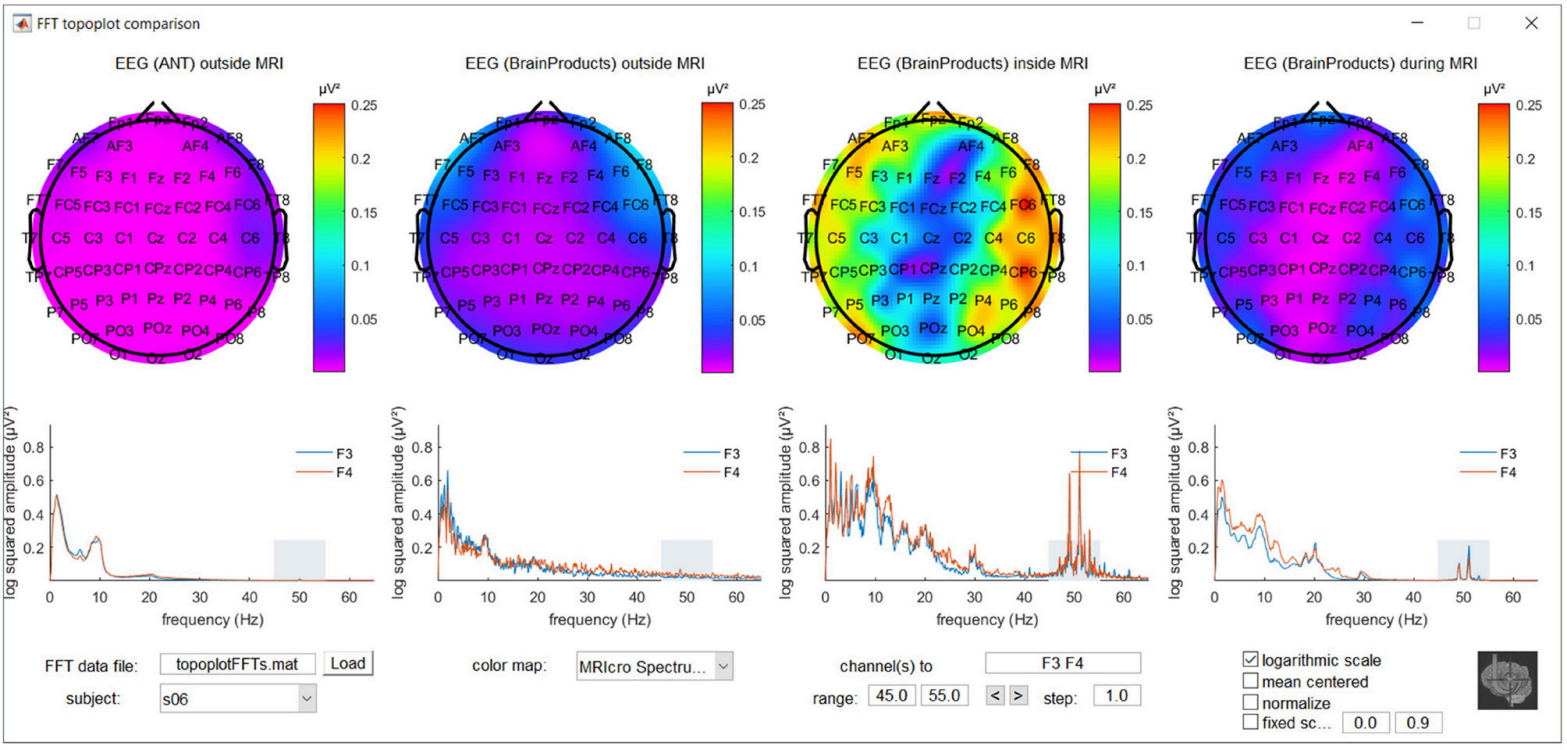
Screenshot of the MATLAB based graphical user interface incorporating the topoplot functionality from EEGLAB, allowing the topographical exploration of fast Fourier transform (FFT) squared amplitudes for a selected frequency range for different recording conditions, with interactive options to selectively view specific frequency ranges and electrode subsets and offering different forms of scaling and color coding. FFT information around the powerline noise frequency of one of the subjects included in the study is shown as an example.

To further explore location and frequency dependent differences between recording conditions, a MATLAB based graphical user interface was developed. It incorporates the topoplot functionality from EEGLAB, allowing the topographical exploration of FFT squared amplitudes for a selected frequency range for the different recording conditions, with interactive options to selectively view specific frequency ranges and electrode subsets and offering different forms of scaling and color coding. The code for this graphical user interface, along with demonstration data, is freely available via MATLAB Central's File Exchange via https://nl.mathworks.com/matlabcentral/fileexchange/67688-frequencytopoplotcomparison under BSD license and a screenshot can be appreciated in Figure 1.

To compare the EEG between recording conditions, a balanced two-way analysis of variance (ANOVA) (as implemented in MATLAB), was used with recording condition as the first factor (levels: [EEG][fMRI], [EEG fMRI]) and electrode as the second factor (multiple levels dependent on the specific comparison). We performed two two-way ANOVAs: one in which we used all electrodes as different levels for the second factor and one in which we took the absolute difference between symmetrically located left and right hemisphere electrodes for the second factor. The statistical threshold was set at a FDR-corrected *p* < 0.05.

Additionally, the EEG during the task ([EEG][fMRI] and [EEG fMRI] recording condition) were qualitatively compared with the EEG during rest ([EEG fMRI]_outside_ and [EEG fMRI]_pre−acquisition_ recording conditions) for the reasons mentioned above.

### 2.3. fMRI

Imaging was performed with a 3 Tesla Philips Achieva equipped with a 32-channel head volume coil (Philips Medical Systems, Best, The Netherlands). Structural scans were acquired according to a T1-weighted 3D turbo-field-echo sequence (repetition time (TR) 9.6 ms, echo time (TE) 4.6 ms, in-plane resolution 0.97 mm, slice thickness 1.2 mm) and whole-brain functional scans consisted of T2* gradient-echo echoplanar images acquired continuously in ascending order (TR 2.0 s, echo time 30 ms, 90° flip angle, 80 × 80 acquisition matrix, 2.75 × 2.75 mm^2^ in-plane resolution, 36 3.75 mm thick axial slices without gap).

Using SPM12, the following preprocessing for each of the 4 runs per subject was performed: conversion from PAR/REC to NIfTI, manually setting the anterior commissure as the origin, realigning the functional scans per subject, slice timing correction for the functional scans, coregistration of the anatomical and the functional scans, segmentation of the anatomical scan, spatial normalization of the functional and the anatomical images into Montreal Neurological Institute (MNI) space. For all steps the default settings were used, except for a larger bounding box [–90 –126 –72; 90 90 108] and voxel sizes of 3 × 3 × 3 mm^3^ for the functional and 1 × 1 × 1 mm^3^ for the structural images during normalization. Full-width at half maximum of the Gaussian smoothing kernel was set to 8 × 8 × 8 mm^3^.

Using SPM12's image calculator, a TSNR image was calculated for every run as $\frac{\mu \left(X\right)}{\sigma \left(X\right)}$, in which *X* is the signal over time, μ(*X*) is its mean and σ(*X*) is its standard deviation (Triantafyllou et al., 2005).

In SPM12, we used a design in which subject (5 independent levels with equal variance) and recording condition (non-independent levels with equal variance: [EEG][fMRI], [EEG fMRI]) were modeled to study the TSNR images. SPM12's intracranial volume mask (mask_ICV.nii) was set as an explicit mask. t-contrasts ([EEG][fMRI] − [EEG fMRI]) and ([EEG fMRI] − [EEG][fMRI]) were calculated. The statistical threshold was set at an uncorrected *p* < 0.001 at the voxel level combined with a family-wise error (FWE) corrected *p* < 0.05 at the cluster level.

Additionally, we investigated various metrics to see variations per tissue type/ratios to see global influences of EEG on TSNR (Liu, 2016; Caballero-Gaudes and Reynolds, 2017; Wald and Polimeni, 2017). To see how the metric changes between the two recording conditions, a simple percentile bootstrap on differences was performed.

Differences in head movement were analyzed by calculating the mean of the instantaneous movement (volume by volume, in mm) per run—obtained from the realignment step in the fMRI preprocessing—using a balanced two-way ANOVA with recording condition as the first factor (levels: [EEG][fMRI], [EEG fMRI]) and run as the second factor (levels: 1–4). The statistical threshold was set at *p* < 0.05.

### 2.4. Tools

All signal processing was performed in MATLAB 9.1.0.441655 (R2016b) (MathWorks, Natick, MA, United States) (RRID:SCR_001622) under Microsoft Windows 10.0.14393 64 bit (Microsoft, Redmond, WA, United States) on a Dell Latitude E7450 (Dell, Round Rock, TX, United States). The software versions used were: ASA 4.6.0.8 (Advanced Neuro Technology (ANT), Enschede, The Netherlands) (RRID:SCR_012867), BrainVision Recorder 1.20.0701 (Brain Products, Gilching, Germany) (RRID:SCR_016331), BrainVision Analyzer 2.1.2.327 (Brain Products, Gilching, Germany) (RRID:SCR_002356), EEGLAB 14.1.1 (Delorme and Makeig, 2004) (RRID:SCR_007292) with toolboxes Libeep EEGLAB plugin 1.13 64bit (RRID:SCR_016334), BVA import/export EEGLAB plugin 1.57 (RRID:SCR_016333), Bergen fMRI Toolbox Plugin for EEGLAB 1.04 (Moosmann et al., 2009) (RRID:SCR_016335), FMRIB toolbox 2.00 (Niazy et al., 2005) (RRID:SCR_005283), ERPLAB 6.1.4 (Lopez-Calderon and Luck, 2014) (RRID:SCR_009574) and SPM12 6906 (Wellcome Trust Centre for Neuroimaging, UCL, London, United Kingdom) (RRID:SCR_007037). Spectrogram color maps are plotted using the MRIcron spectrum color map equalized for color and lightness using the CIE76 formula (Cyril Pernet, University of Edinburgh, Edinburgh, United Kingdom) (RRID:SCR_016715). TSNR in the different tissue compartments was calculated using the spmup_temporalSNR.m function from the SPM U+ toolbox (Cyril Pernet, University of Edinburgh, Edinburg, United Kingdom) (RRID:SCR_016743).

## 3. Results

### 3.1. EEG

**Figure 2:**
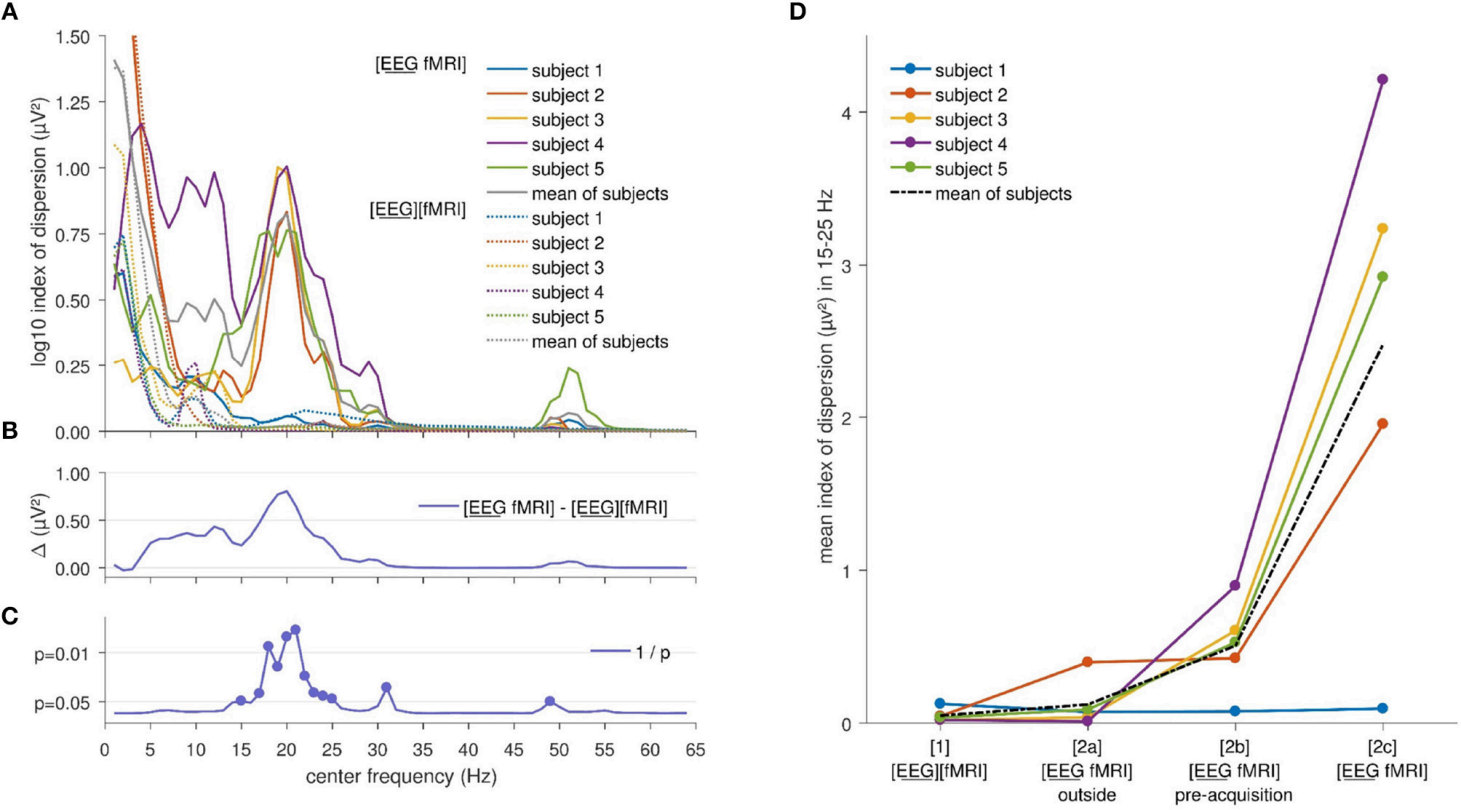
Electroencephalography (EEG) analysis: **(A)** Comparison of the logarithm (base 10) of the index of dispersion over all electrodes of the squared amplitude of the fast Fourier transform (FFT) between the separately ([EEG][fMRI]) and simultaneously ([EEG fMRI]) recorded EEG during the task, per frequency range. For every data point the mean squared amplitude of the FFT for a frequency interval of 2 Hz around the center frequency was calculated. **(B)** Mean difference Δ of the logarithm (base 10) of the index of dispersion of the squared amplitude of the FFT between the separately ([EEG][fMRI]) and simultaneously ([EEG fMRI]) recorded EEG. **(C)** Uncorrected p values as function of frequency of the difference shown in the previous panels. **(D)** Mean index of dispersion in the interval 15–25 Hz for the different recording conditions.

Index of dispersion analysis of *A*^2^(*f*), the squared amplitude of FFT, showed no significant differences at the FDR-corrected *p* < 0.05 between the [EEG][fMRI] and [EEG fMRI] recording conditions although it was systematically higher in the simultaneous measurement ([EEG fMRI]) compared to the separate recording condition (see Figures 2B,C). Furthermore, in the frequency range 15–25 Hz (Figures 2A,B) a clear peak was found in the difference between the two recording conditions and therefore, we will focus on the average value of the squared amplitudes of FFT within this frequency interval in the remainder of the paper.

**Figure 3:**
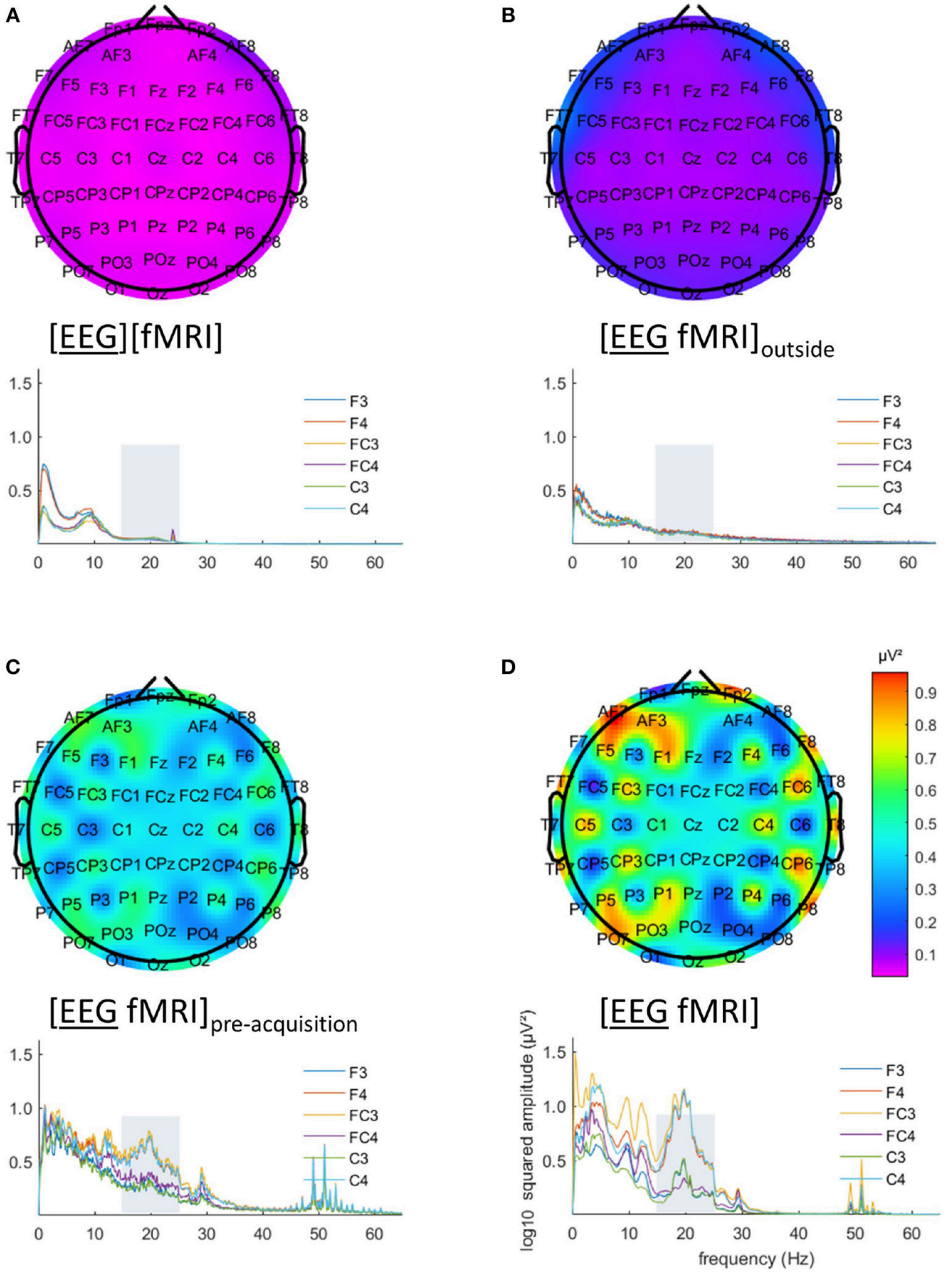
Electroencephalography (EEG) analysis: topographic plots of the logarithm (base 10) of the mean squared amplitude of the fast Fourier transform (FFT) in the frequency range 15–25 Hz (top) and frequency × squared amplitude of the FFT curves of example electrodes F3/4, FC3/4 and C3/4 (bottom) for the mean of all five subjects, for the different recording conditions, as captured from our graphical user interface: EEG **(A)** recorded separately during the task ([EEG][fMRI]), **(B)** recorded in the scanner control room during rest ([EEG fMRI]_outside_) **(C)** recorded inside the scanner during rest before image acquisition ([EEG fMRI]_pre−acquisition_) and **(D)** recorded inside the scanner during the task with image acquisition ([EEG fMRI]).

EEG analysis of the 15–25 Hz frequency range showed a particular pattern with a left-right difference and alternating effects on adjacent electrodes, where relatively low and similar values for left and right regions are to be expected. This pattern is clearly visible in Figures 3C,D. This pattern was present in every single subject.

Figure 2D shows the mean index of dispersion for the frequency range 15–25 Hz for the measurements outside the scanner in the [EEG][fMRI] (0.05 μV^2^) and the [EEG fMRI]_outside_ recording condition (0.12 μV^2^). It also shows an increased index of dispersion for measurements inside the scanner in the [EEG fMRI]_pre−acquisition_ (0.51 μV^2^) which is further increased in the [EEG fMRI] recording condition (2.48 μV^2^).

Analysis of the mean squared amplitude in the frequency range 15–25 Hz revealed a significant main effect of condition [[EEG][fMRI] (0.12 μV^2^) vs. [EEG fMRI] (3.08 μV^2^); *F*_(1, 480)_ = 284.1, *p* < 10^-49^], a significant main effect of electrode [*F*_(59, 480)_ = 3.63, *p* < 10^–14^] and a significant interaction effect [*F*_(59, 480)_ = 3.61, *p* < 10^–14^].

Analysis of the absolute difference of the mean squared amplitude in the frequency range 15–25 Hz between symmetrically located left and right electrodes (e.g., F3-F4, PO7-PO8, …) revealed a significant main effect of condition [[EEG][fMRI] (0.12 μV^2^) vs. [EEG fMRI] (3.25 μV^2^); *F*_(1, 208)_ = 179.7, *p* < 10^–29^], but no main effect of electrode pair [*F*_(25, 208)_ = 1.15, *p* = 0.29] or interaction effect [*F*_(25, 208)_ = 1.11, *p* = 0.34].

To identify in which electrodes and to what degree there were differences, an ANOVA analysis with factors recording condition (levels: [EEG][fMRI], [EEG fMRI]) and electrode (levels: left hemispheric, right hemispheric) was carried out, repeated for every symmetrical non-midline electrode pair (e.g., F3 & F4, PO7 & PO8, …). This showed a main effect of condition for all electrode pairs [*F*_(1, 16)_ ≥ 6.21, FDR-corrected *p* ≤ 0.05]. A main effect of electrode pair and an interaction effect were present with a gradient from central to peripheral and became significant when electrodes were two or more distances away from the Cz (Table 1). Electrodes over the left hemisphere belonging to the first amplifier showed lower FFT squared amplitude values than electrodes over the right hemisphere (one-sample *t*-test of the left–right difference for the mean squared amplitude of FFT over subjects: FDR-corrected *p* < 0.05; mean difference 4.53 μV^2^). For electrodes belonging to the second amplifier this was the other way around (FDR-corrected *p* < 0.05; mean difference 5.12 μV^2^).

**Table 1:** Electroencephalography (EEG) analysis of the difference in mean squared amplitude of the fast Fourier transform (FFT) between corresponding electrode pairs: results of the ANOVA analysis repeated for every symmetrical non-midline electrode pair evaluating the main effect of recording condition (levels: recorded separately from functional magnetic resonance imaging (fMRI) ([EEG][fMRI]), recorded simultaneously with fMRI ([EEG fMRI])), the main effect of electrode (levels: left hemispheric, right hemispheric) and their interaction effect.

	**Electrode**	**Main effect of**	**Main effect of**	**Interaction**
**Amplifier**	**pair**	**electrode pair**	**recording condition**	**effect**
	FC1-FC2	**0.003**	0.546	0.533
	CP1-CP2	**0.003**	0.641	0.652
	F3-F4	**0.003**	**0.019**	**0.019**
	C3-C4	**0.003**	**0.019**	**0.018**
	P3-P4	**0.002**	**0.025**	**0.025**
1			
	O1-O2	**0.007**	0.067	0.065
	FC5-FC6	**0.014**	**0.019**	**0.020**
	CP5-CP6	**0.009**	**0.014**	**0.015**
	Fp1-Fp2	**0.014**	**0.027**	**0.027**
	F7-F8	**0.002**	**0.007**	**0.007**
	P7-P8	**0.001**	**0.006**	**0.007**
	T7-T8	**0.003**	**0.012**	**0.013**
				
	C1-C2	**0.013**	0.614	0.610
	AF3-AF4	**0.019**	0.080	0.082
	F1-F2	**0.024**	0.076	0.076
	FC3-FC4	**0.017**	0.051	0.051
	CP3-CP4	**0.015**	0.036	0.035
	P1-P2	**0.020**	0.058	0.058
2	
	PO3-PO4	**0.012**	0.037	0.037
	F5-F6	**0.010**	**0.020**	**0.019**
	C5-C6	**0.009**	**0.018**	**0.017**
	P5-P6	**0.009**	**0.020**	**0.020**
	AF7-AF8	**0.001**	**0.003**	**0.003**
	PO7-PO8	**0.001**	**0.005**	**0.005**
	FT7-FT8	**0.001**	**0.003**	**0.003**
	TP7-TP8	**0.001**	**0.005**	**0.005**

*p-values are uncorrected but those surviving a false discovery rate (FDR) correction at alpha = 0.05 are shown in bold. Electrode pairs are sorted according to their distance from Cz*.

The EEG in the [EEG fMRI]_outside_ recording condition did not show the pattern found in the [EEG fMRI] recording condition, as depicted in Figure 3B. Figure 3A shows the EEG of the separate [EEG][fMRI] for comparison.

The EEG in the [EEG fMRI]_pre−acquisition_ recording condition did show the same pattern as in the [EEG fMRI] recording condition. This pattern had the same direction of effect, but was less pronounced (Figure 3C).

Figures 3C,D also clearly show additional squared amplitude peaks in a relatively broad region around 50 Hz for all measurements inside the scanner ([EEG fMRI]_pre−acquisition_, [EEG fMRI]) compared to outside the scanner ([EEG][fMRI], [EEG fMRI]_outside_). This is suggestive of electrical interference from nearby equipment.

To exclude a possible effect of the preprocessing pipeline (Ritter et al., 2007), we randomly selected one subject's full dataset, preprocessed it with BrainVision Analyzer instead of the MATLAB based pipeline detailed above (gradient artifact subtraction, down sampling, bandpass filtering and cardioballistic artifact subtraction) and visually analyzed it. Results were similar for both pipelines.

**Figure 4:**
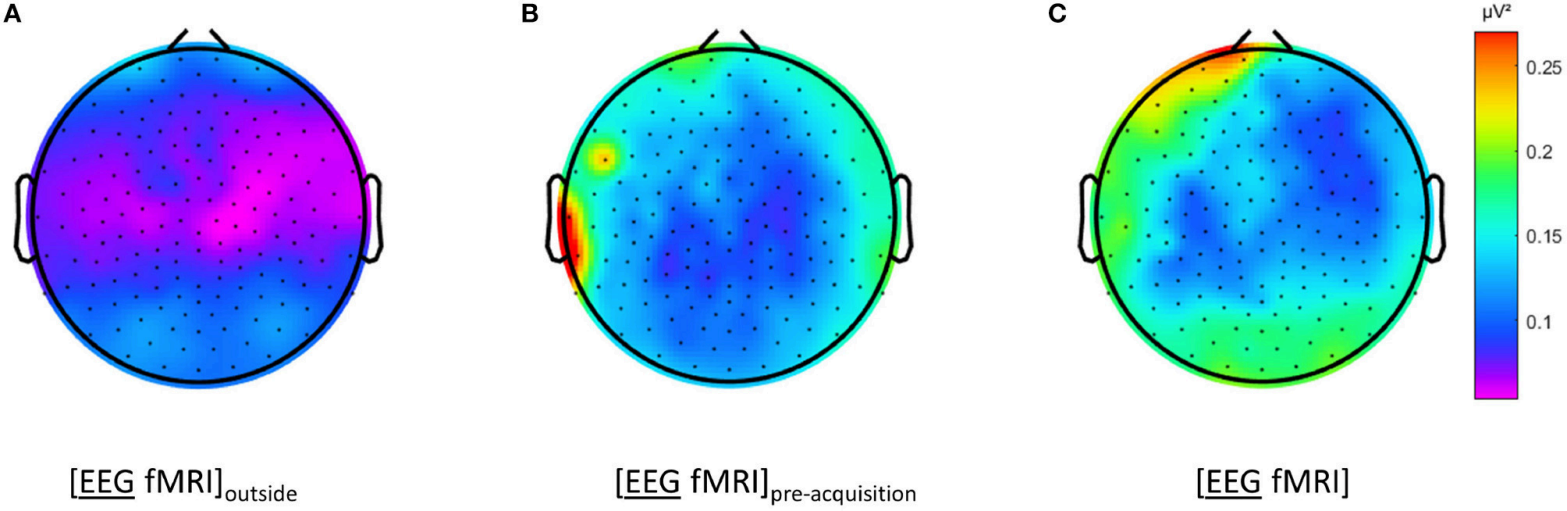
Electroencephalography (EEG) analysis: topographic plots of the logarithm (base 10) of the mean squared amplitude of the fast Fourier transform (FFT) in the frequency range 15–25 Hz for a single subject during different recording conditions, as captured from our graphical user interface: during rest EEG **(A)** recorded in the scanner control room ([EEG fMRI]_outside_), **(B)** recorded inside the scanner before image acquisition and **(C)** recorded inside the scanner during image acquisition ([EEG fMRI]).

To verify whether the EEG effects were only present in our specific set-up, we qualitatively inspected for regional inhomogeneities in EEG data recorded during rest, using loose electrodes and other EEG caps using the same amplifier and scanner. Additionally EEG data during rest recorded with a 256 channel Geodesic EEG System (Electrical Geodesics Inc., Eugene, OR, United States) and a different 3 Tesla Philips Achieva scanner, were visually compared. In one session, subsequent recordings were made outside the MRI scanner, inside the scanner before image acquisition and during image acquisition. Inhomogeneities over the scalp and over frequencies was demonstrated inside the scanner, both before and during image acquisition, but in a different pattern that was mostly less pronounced. See Figure 4 for an example of the dataset with 256 channels.

### 3.2. fMRI

**Figure 5:**
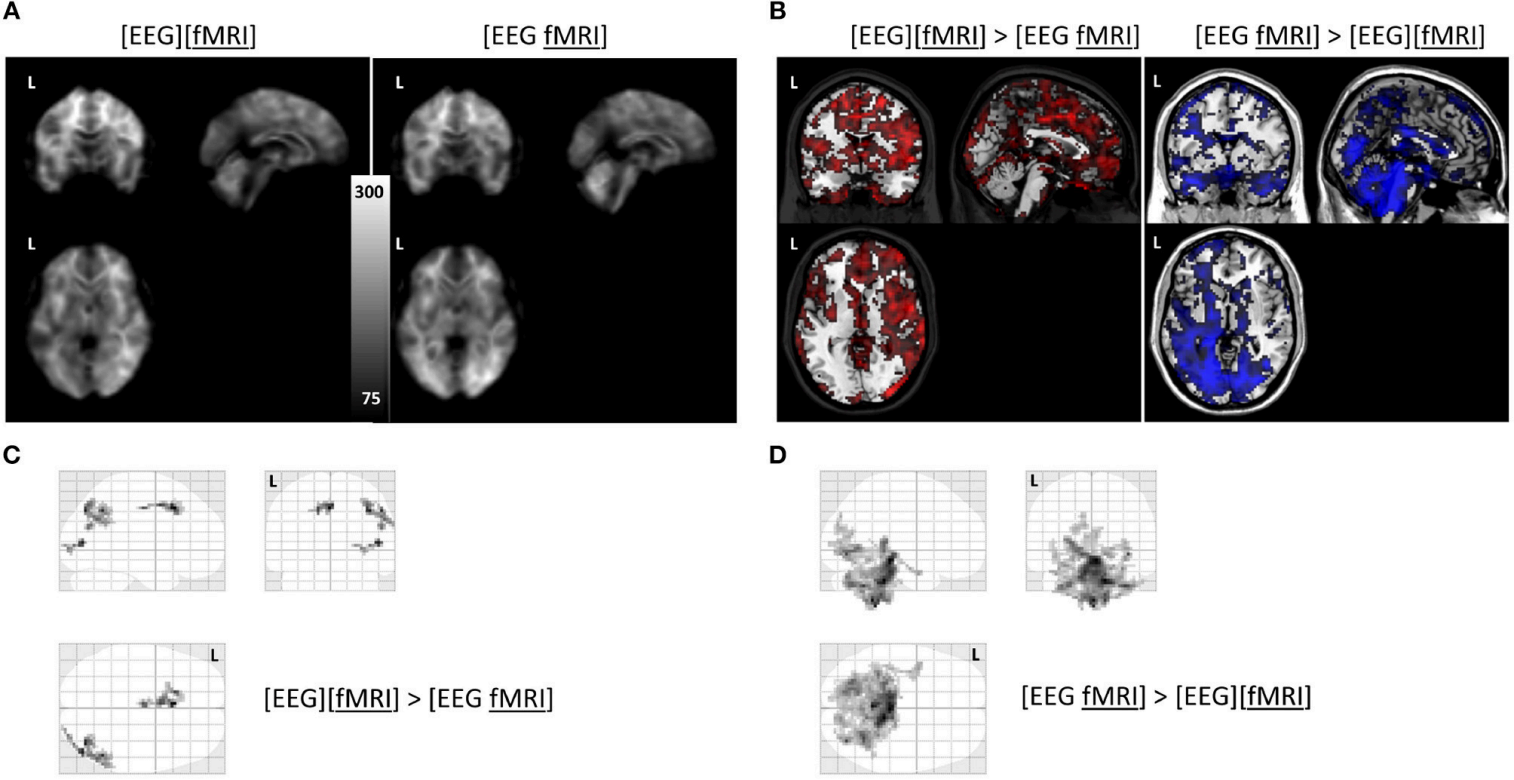
Functional magnetic resonance imaging (fMRI) analysis: **(A)** average temporal signal to noise ratio (TSNR) maps in the separate and simultaneous recording condition; **(B)** unthresholded T-map overlayed on brain template ch2 available in MRIcron. Positive T-values are shown in red (corresponding to TSNR values which are bigger in the separate compared to the simultaneous condition; negavitve T-values are shown in blue (corresponding to the reverse contrast). **(C)** glass brain view of the clusters with a higher TSNR in the recording condition without electroencephalography (EEG) electrodes ([EEG][fMRI]) compared to the recording condition with EEG electrodes ([EEG fMRI]) and **(D)** the reverse comparison. L = left.

Global changes in TSNR per tissue type/ratios were assessed with a simple percentile bootstrap method. We could not find any difference in any of these global metrics (all percentiles were in the range 40–60%). However, when evaluating the local changes, we found that TSNR was higher in the [EEG][fMRI] recording condition in right occipital and parietal regions and in the cingulum, compared to the [EEG fMRI] recording condition (see Figures 5A–C and Table 2).

**Table 2:** Functional magnetic resonance imaging (fMRI) analysis: clusters with a higher temporal signal to noise ratio (TSNR) when comparing the recording condition without electroencephalography (EEG) electrodes ([EEG][fMRI]) to the recording condition with EEG electrodes ([EEG fMRI]).

**[EEG][fMRI]** > **[EEG fMRI]**						
**Cluster**	**Cluster size**	**Peak**	**Peak**	**x**	**y**	**z**
**p_FWE_**	**(voxels)**	**p_FWE_**	**T**	**(mm)**	**(mm)**	**(mm)**
< 0.001	113	0.007	6.48	–3	18	42
		0.18	5.40	–15	18	42
		0.19	5.39	–3	3	45
< 0.001	141	0.035	5.95	39	–75	39
		0.07	5.71	51	–57	39
		0.40	5.07	54	–63	30
0.040	40	0.04	5.91	48	–78	6
		0.71	4.70	39	–87	3
		0.73	4.68	30	–93	0
< 0.001	2,487	< 0.001	9.45	3	–30	–12
		< 0.001	9.14	12	–36	–33
		< 0.001	8.78	0	–48	–54
< 0.001	128	0.001	7.20	36	–75	–6
		0.13	5.52	12	–81	–6
		0.59	4.84	33	–81	6
0.012	52	0.57	4.86	–9	–84	24

*p_FWE_: family-wise error (FWE) corrected p value, x y z coordinates in Montreal Neurological Institute (MNI) space. The table shows at most 3 local maxima within each cluster at least 10 mm apart*.

TSNR was higher in the [EEG fMRI] recording condition in a largely symmetrical broad posterior and caudal region, including inferior occipital cortex, diencephalon and brainstem compared to the [EEG][fMRI] recording condition (see Figures 5A,B,D and Table 2).

Overall, instantaneous (volume to volume) head movement during fMRI was minimal (median 0.065 mm; interquartile range 0.068 mm; maximum 0.72 mm). Mean instantaneous head movement per run was comparable across runs (*p* = 0.96), but was smaller in the [EEG fMRI] recording condition (mean 0.0732 mm; interquartile range 0.021 mm) compared to the [EEG][fMRI] recording condition (mean 0.092 mm; interquartile range 0.048 mm) (*p* < 0.018). There was no interaction effect (*p* = 0.91).

## 4. Discussion

Quality comparison of EEG data has been investigated between simultaneous and separate recordings by visual inspection (Laufs et al., 2003; Foged et al., 2017) and simulation studies (Ihalainen et al., 2015). More quantitative spectral approaches for comparing measurements have been described. For example comparing spectral differences between EEG measured inside the scanner with and without image acquisition during periodic fMRI recordings (Allen et al., 2000; Laufs et al., 2003; Ritter et al., 2007), and EEG measured inside the scanner during image acquisition and outside the scanner (Allen et al., 1998; Bénar et al., 2003). These studies revealed acceptable, but quantifiable differences.

In this paper an approach is presented to quantify the amount of regional differences in signal interference between EEG and fMRI when measured simultaneously based on TSNR for imaging data and spectral data for EEG. Signal interference was substantial in both directions, even after optimized conventional corrections and artifact removal procedures. Our study and method adds spectral and topographical EEG information to the literature. Additionally, comparisons across EEG measurements were made outside the scanner, inside the scanner before acquiring images and inside the scanner during image acquisition instead of just two conditions. A graphical user interface was developed to efficiently explore possible differences in the frequency domain and regional differences. Although these effects were already quantifiable in the scanner prior to image acquisition, they were not identified by visual inspection of the on-line measurement at the time of recording.

Not only could clear differences in power spectra between measurements inside and outside the MRI scanner be demonstrated, the difference effects in our set-up were inhomogeneous over electrodes (left vs. right, central vs. peripheral) and frequency dependent. The type of differences found were consistent across all subjects. Although larger sample sizes are desirable, the current EEG findings are consistent across all subjects, making it highly unlikely that one would arrive at a different conclusion with a larger sample.

It is evident from our study that recording a quality measurement of the acquisition system outside the MRI scanner alone, e.g., in the control room, is not sufficient. Since effects are amplified during image acquisition, recording a quality measurement inside the scanner prior to scanning might not suffice either. The fact that effects are present inside the scanner without image acquisition—although to a lesser degree than during image acquisition—does not make recordings with intermittent image acquisition a failsafe alternative.

Note that since we re-referenced the data to the average reference before limiting our analysis to the common set of electrodes between both caps, the average reference for the different recording systems is slightly different. Performing re-referencing to the average after using only the common set of electrodes between the two caps, would still lead to a slightly different reference, as the positions of the electrodes in both caps are not perfectly the same. We opted to accept this as one of the many inherent differences between the recording systems used. To exclude a significant influence of this choice, the above mentioned EEG analyses were also performed on the data re-referenced to the average reference using only the common set of electrodes. This did not change the significance or the magnitude of any of the results.

We were not able to statistically compare impedance levels between the recording systems, as these were not saved to disk for one of the systems. A difference in impedances as a unique explanation for the described differences is not expected, given that we used the same predefined impedance criteria before acquisition on both systems and that pronounced effects could be demonstrated between measurements outside and inside the scanner within the same system.

Our recording system consists of two amplifiers. The differences among the electrodes are not segregated by amplifier, as left and right symmetrically located electrodes show the difference effect and always belong to the same amplifier. Per amplifier, there was a predictable effect, which might correspond to the cyclical interference described in Ihalainen et al. (2015).

The amount of auditory equipment-related background noise was not controlled for in the different conditions. It was, however, larger for the [EEG fMRI] condition compared to the [EEG][fMRI] condition before image acquisition, and even larger during image acquisition. Nonetheless, it is unlikely that this is the source of the differences observed in EEG, as this would not explain the alternating pattern observed in Figure 2D. If it were the source of the differences, it would have resulted in a similar pattern to that observed in datasets recorded with different hardware, which was not the case.

An interaction between the sequence or pace of the task and the frequencies in which we found the most pronounced differences is not supported by the timings in the task. It is also unlikely given the above mentioned alternating pattern and the fact that it was also present inside the scanner before the task/image acquisition.

Instead, it is possible that an interaction between the magnetic fields of the MRI (static field and gradient switches) and cap factors (distance of the electrodes from the reference electrode, orientation of the electrode with respect to the magnetic fields, cable positioning on the cap and in the ribbon cable) is having an effect on the amplitude and specific frequencies that are seen to be affected. Which of these factors might be the most important is highly speculative and not the specific goal of this study.

A limitation in this study is that we did not record EEG data in the scanner control room and inside the scanner bore before image acquisition during the same covert attention task. Another limitation is that we didn't use the same recording equipment in the [EEG][fMRI] condition as in the [EEG fMRI] condition. However, in our experience with the paradigm, the size of the EEG effects induced by this task are not of the magnitude that it means our results would be invalidated. Moreover, within the rest condition, we could demonstrate differences between recordings in the control room and inside the scanner before image acquisition, confirming that the MRI is a necessary factor to explain our results.

A number of similar-sized studies have already investigated the effects of dense array EEGs on MRI and fMRI signals. Typically, anatomical images of scalp and skull are severely affected up to 15 mm due to susceptibility artifacts. The effects are more subtle for the cortex and subcortex. In specific regions such as the occipital region (Luo and Glover, 2012; Ihalainen et al., 2015; Klein et al., 2015) effects have been described on measures such as cortical thickness, surface area, volume and signal to noise ratio. The effect on BOLD activation for fMRI is thought to be non-significant, although reduced signal to noise ratios have been reported (Luo and Glover, 2012; Klein et al., 2015). This claim is often made based on paradigms with a relatively strong BOLD response. We argue that for weaker effects this might not be the case. fMRI TR duration is considered of limited influence (Foged et al., 2017). The regional effects found in this study are somewhat different from these described in the literature (Luo and Glover, 2012; Ihalainen et al., 2015; Klein et al., 2015), underlining that for every individual set-up a quality test is advisable. Removing image distortions in the preprocessing pipeline, that are induced by the presence of EEG hardware during the functional scans should be considered based on quantitative comparison of its magnitude, e.g., as presented here.

Although one might intuitively expect subjects to move a little more when wearing an EEG cap, as this recording condition could be more uncomfortable, the opposite was true. Since all subjects participated in the simultaneously recorded experiment after they had already participated in the separately recorded experiment, a training effect could possibly partly explain the difference in movement. We speculate that aside from physically hampering head movement, the EEG cap provides sensory feedback on head movement, which could have helped reduce movement.

While it is evident that some of the effects as described here are likely specific to the experimental set-up used (e.g., the alternating EEG pattern), the appearance of (different) EEG inhomogeneities in recordings with different hardware could be documented as well.

Based on our findings, performing a quality control test before starting a combined EEG-fMRI measurement to ensure that there are no uncorrectable adverse effects present in regions and frequencies that are of special interest to the research question at hand seems warranted. The EEG tool we provide in this manuscript can visualize the squared amplitudes of the fast Fourier transform (FFT) per frequency, or any other quantitative measure, between different recording conditions such as e.g., separately ([EEG][fMRI]) and simultaneously ([EEG fMRI]) recorded EEG per frequency. This allows an easy interactive assessment of those frequencies in which substantial changes are observed. If this is the case in frequency bands of interest, one has to take this into account when considering simultaneous [EEG fMRI] experiments. The same holds true when comparing global and local changes in TSNR for fMRI when considering simultaneous [EEG fMRI] experiments.

## Data Availability

The pre-processed data underlying the findings are available at Zenodo, doi: 10.5281/zenodo.1507643.

## Author Contributions

MS performed research, analyzed data and drafted the paper. PD data analysis revision. RP, RV, and PD paper revision.

### Conflict of Interest Statement

The authors declare that the research was conducted in the absence of any commercial or financial relationships that could be construed as a potential conflict of interest.

## Footnotes

^1^To be unequivocal about which measurement modality is the subject of the sentence, be it the EEG or the fMRI component of the recording condition, this component is additionally underlined.
